# Reduced activity and connectivity of left amygdala in patients with schizophrenia treated with clozapine or olanzapine

**DOI:** 10.1007/s00406-018-0965-4

**Published:** 2018-12-11

**Authors:** Daniela Mier, Frederike Schirmbeck, Gabriela Stoessel, Christine Esslinger, Franziska Rausch, Susanne Englisch, Sarah Eisenacher, Lieuwe de Haan, Andreas Meyer-Lindenberg, Peter Kirsch, Mathias Zink

**Affiliations:** 1 Department of Clinical Psychology, Central Institute of Mental Health, Medical Faculty Mannheim/University of Heidelberg, Mannheim, Germany; 2 Department of Psychology, University of Konstanz, Constance, Germany; 3 Department of Psychiatry, Academic Medical Centre, University of Amsterdam, Amsterdam, The Netherlands; 4 Department of Psychiatry and Psychotherapy, Central Institute of Mental Health, Medical Faculty Mannheim/University of Heidelberg, Mannheim, Germany; 5 Department of Psychiatry, Arkin Institute for Mental Health, Amsterdam, The Netherlands; 6 Department of Psychiatry, Psychotherapy and Psychosomatics, District Hospital Ansbach, Ansbach, Germany

**Keywords:** Schizophrenia, Obsessive–compulsive, Amygdala, fMRI, Antipsychotic

## Abstract

Obsessive–compulsive symptoms (OCS) in patients with schizophrenia are a common co-occurring condition, often associated with additional impairments. A subgroup of these patients develops OCS during treatment with second-generation antipsychotics (SGAs), most importantly clozapine and olanzapine. So far, little is known about possible neural mechanism of these SGAs, which seem to aggravate or induce OCS. To investigate the role of SGA treatment on neural activation and connectivity during emotional processing, patients were stratified according to their monotherapy into two groups (group I: clozapine or olanzapine, *n* = 20; group II: amisulpride or aripiprazole, *n* = 20). We used an fMRI approach, applying an implicit emotion recognition task. Group comparisons showed significantly higher frequency and severity of comorbid OCS in group I than group II. Task specific activation was attenuated in group I in the left amygdala. Furthermore, functional connectivity from left amygdala to right ventral striatum was reduced in group I. Reduced amygdala activation was associated with OCS severity. Recent literature suggests an involvement of an amygdala–cortico–striatal network in the pathogenesis of obsessive–compulsive disorder. The observed differential activation and connectivity pattern of the amygdala might thus indicate a neural mechanism for the development of SGA-associated OCS in patients with schizophrenia. Further neurobiological research and interventional studies are needed for causal inferences.

## 1. Introduction

Psychiatric comorbidities are common among patients with schizophrenia. One of the foremost comorbid conditions are obsessive–compulsive symptoms (OCS), reported in 30% of cases, with about 13% fulfilling the criteria of an obsessive–compulsive disorder (OCD) [1, 2]. Co-occurring OCS results in a lower quality of life [3], more severe depressive symptoms [4], higher rates of suicidality [5] and an unfavorable prognosis [6, 7].

The clinical presentation of OCS in schizophrenia is diverse with onset prior to, simultaneously with or subsequent to the onset of psychosis. This heterogeneity suggests multiple interacting pathways [8]. For the subgroup of patients, who develop OCS subsequent to their first psychotic episode and initiation of antipsychotic treatment, increasing evidence strongly suggests a “pro-obsessive” effect of certain second-generation antipsychotics (SGAs), especially clozapine [9, 10] and olanzapine [11]. Clozapine, and similarly olanzapine, exerts its effects via a relatively low affinity to dopamine D_2_ receptors combined with strong antagonism at 5-HT_1C_, 5-HT_2A_ and 5HT_2C_ receptors [12, 13]. In contrast, predominantly dopaminergic SGAs such as amisulpride [14] or the partial dopaminergic/serotonergic agonist aripiprazole [15] are two substances that rather seem to have a beneficial or at least neutral effect on OCS [16–20]. Hence, differences in receptor binding profiles of clozapine/olanzapine vs. amisulpride/aripiprazole might explain diverging effects on co-occurring OCS [21]. The assumption that SGAs aggravate OCS via an antiserotonergic mechanism also seems plausible, because treatment with selective serotonin reuptake inhibitors (SSRIs) has been proven effective in the treatment of OCD [22]. Furthermore, CBT treatment of OCD exerts a serotonergic normalization and several clinical trials revealed positive effects of serotonergic antidepressants for comorbid OCD in schizophrenia [23].

The neural mechanisms of how antipsychotics change brain functioning are poorly understood. Röder et al. reviewed literature on antipsychotic influence on the blood-oxygenation-level-dependent (BOLD)-signal and suggested that functional magnetic resonance imaging (fMRI) can be a useful approach to provide information about differential drug effects [24]. In a prior study, we investigated differential effects of SGAs on OCS in schizophrenia by comparing patients treated with clozapine or olanzapine to a group treated with aripiprazole or amisulpride [25, 26]. FMRI analyses showed aberrant orbitofrontal cortex (OFC) activation during a flanker task in the clozapine/olanzapine group. OFC activation mediated the association between SGA treatment and co-occurring OCS [27]. To the best of our knowledge, four other fMRI studies investigated neural correlates of OCS in schizophrenia, but did not specifically focus on OCD-related brain regions, nor did they account for possible underlying pharmacodynamic mechanisms [28–30].

Apart from the fronto-striato-thalamocortical (CSTC) circuitry, which is known to be involved in OCD pathogenesis [31, 32], recent findings proposed to extend research to limbic regions [33–35], and highlighted the role of the amygdala,[36, 37].

In their review, Wood and Ahmari [37] discussed the potential role of a corticolimbic-ventral striatum network, extending the traditional OCD model with an important aspect, namely affective dysregulation. This circuit connecting frontal and limbic brain regions, specifically the amygdala with the ventral striatum plays a particularly important role in the emotional appraisal of situations and the generation of emotional responses and reward-based behaviors. The circuit has been found altered in OCD explaining increased anxiety and repetitive behaviors [34, 37].

Accordingly, a number of fMRI studies reported increased amygdala activation [38] and enhanced amygdala-prefrontal connectivity during emotion recognition tasks [39], whereas others showed attenuated amygdala responsivity to emotional stimuli in OCD patients relative to healthy controls [40, 41].

Differences in amygdala activity and corticolimbic connectivity during emotion processing have also been found in patients with schizophrenia [42, 43]. Meta-analytic findings suggest hypoactivation of the amygdala in response to emotional facial expressions compared to healthy controls [44], whereas more recent studies reported hyperactivation in response to neutral facial expressions [45, 46].

Hence, aberrant amygdala activation and connectivity to regions of the corticolimbic-ventral striatum network during emotion processing might indicate a neural correlate of both OCD and schizophrenia and might play a role in the co-occurrence of the two disorders. However, additive effects and modulations due to therapeutic interventions seem possible. Accordingly, pharmacological treatment affects brain activation involved in emotional processing [24, 47, 48]. Effects of antipsychotic agents should be considered when investigating OCS-related aberrations in brain activation in schizophrenia.

The aim of the present study was to investigate whether SGAs with different pharmacodynamic profiles differentially affect functioning of brain regions known to be involved in emotional processing. We assumed to find differences in brain activation and connectivity especially of the amygdala between SGA groups. On a secondary level, we intended to investigate associations between task-specific activation and the severity of OCS.

## Method

### Study design and participants

This neuroimaging approach was part of a multimodal assessment [25, 26]. Patients were divided into two groups, those with an inherent antiserotonergic profile (group I: olanzapine and clozapine) and those with a primarily dopaminergic treatment profile (group II: amisulpride and aripiprazole) [15, 49]. As described earlier [27], participants were aged 18–60 years, diagnosed with a schizophrenia spectrum disorder according to DSM-IV-TR, received stable monotherapy with clozapine, olanzapine, amisulpride or aripiprazole and showed stable psychopathology over a period of at least 2 weeks with constant severity scores in psychosocial functioning (PSP) and the Positive and Negative Syndrome Scale (PANSS). Exclusion criteria included a history of alcohol or drug addiction or current treatment with antidepressants (except for reboxetine and bupropion—substances without marked serotonergic effects). Benzodiazepine intake was no exclusion criteria, but only one patient was prescribed clonazepam on demand. The investigation was approved by the ethical committee of the University of Heidelberg (no. 2008-235N-MA) and performed in agreement with the guidelines of good clinical practice. All participants provided written informed consent prior to study inclusion.

### Clinical assessment

Sociodemographic and clinical variables were assessed using questionnaires and structured clinical interviews by a trained and certified rater (FS). The Yale–Brown Obsessive–Compulsive Scale (YBOCS) was applied to assess OCS severity, which has been validated in schizophrenia populations [50, 51]. The YBOCS allows the rating of compulsions and obsessions on 5-point Likert scales (0–4), yielding subtotal scores ranging from 0 to 20. According to the interpretation guidelines of the original authors [52], total scores of ≤ 7 are likely to be subclinical, whereas scores of ≥ 8 are likely to represent at least a mild case of OCD.

In addition, the Hamburger Zwangsinventar (HZI) was applied as a self-rating questionnaire to measure the presence of obsessions and different types of compulsions. The severity of psychotic symptoms was rated with the PANSS positive, negative and general psychopathology subscale. Subdomains of negative symptoms were further explored with the five subscales of the Scale for the Assessment of Negative Symptoms (SANS). Comorbid depressive symptoms were rated with the Calgary Depression Scale for Schizophrenia (CDSS). General and social functioning was assessed with the Personal and Social Performance Scale (PSP).

### Functional MRI

To elicit amygdala response, we used the classical implicit emotion recognition face-matching paradigm of Hariri et al. [53]. In this task, participants see three items on a screen: either faces showing emotional states such as anger or fear in the experimental condition or geometrical figures in the control condition. A reference item is shown at the top and two items for comparison left and right below the target item. The task is to indicate which of the two comparison items is identical to the target item. Each face and object was presented for 5 s in an A–B block design. Each block lasted around 30 s with a total experimental duration of 4.5 min. The task was presented with presentation (neurobehavioral systems) and responses were given via button press (current design).

### Data acquisition and analyses

Sociodemographic characteristics and clinical variables at baseline were compared between groups using parametric Student *t* test and *χ*^2^ test. In case the assumption of normal distribution was violated, non-parametric Mann–Whitney *U* test was applied. Effect-sizes were calculated for between-group differences using Cohens’ *d* for normally distributed and Rosenthals’ *r* for non-normally distributed data. Statistical analyses were performed using the Statistical package for Social Sciences (SPSS version 24.0, Chicago, IL, US), assuming a two-sided significance levels of *α* < 0.05.

Functional imaging data was acquired with a 3 T Siemens Tim TRIO (Siemens Erlangen). An echo-planar imaging (EPI) sequence was used with the following parameters: 28 axial slices, field of view 19.2 cm, matrix 64 × 64, voxel size 3 × 3 × 5 mm^3^, repetition time 2000 ms, echo time 30 ms. Scans were acquired in descending order. 134 scans were acquired for the face-matching task. The first four volumes were discarded to account for saturation effects.

Functional imaging data was analyzed using SPM8 (http://www.fil.ion.ucl.ac.uk/spm/software/spm8/). Pre-processing involved realignment, slice time correction, normalization to the standard MNI-EPI-template (Montreal Neurological Institute [MNI] EPI template) with resampling to an isotropic 3 × 3 × 3 mm voxel size and smoothing with a 9 mm full-width at half-maximum Gaussian filter.

To estimate individual neural activity, the general linear model (GLM) was applied to the BOLD-signal change. BOLD changes for each condition (faces, geometrical shapes) were modeled as a convolution of the canonical hemodynamic response function with a box-car function of the corresponding condition. Additionally, head movement was taken into account by means of six regressors (three translations, three rotations) obtained from realignment. For functional connectivity analyses with the left amygdala (seed region), eigenvariate time series were extracted from this region, and used as an additional regressor in the second GLM analysis. Further, eigenvariate time series from white matter and cerebrospinal fluid were extracted and used as covariates. To avoid confounding effects of task activation, amygdala’s eigenvariates were calculated after task activations were regressed out of the data in the first GLM.

For activity analyses of the contrast faces > geometrical figures, second-level group statistics were conducted by one-sample and two-sample *t* tests, and regression analyses. Second-level connectivity analyses were achieved with two-sample *t* test. Significance threshold at the voxel level was set to *p* < 0.05 FWE corrected, *k* = 10 for whole brain analyses. In addition, region of interest (ROI) analyses for activity differences were conducted for left and right amygdala. Furthermore, to investigate differences between groups in connectivity of the amygdala with the corticolimbic-ventral striatal circuitry, we applied ROIs for left and right ventral striatum. Masks were taken from the Wake Forest University (WFU)-Pickatlas. The ROI of the left amygdala was also taken for eigenvariate extraction for functional connectivity. Significance threshold for the ROI analyses at the voxel level was set to *p* < 0.05, small volume corrected (svc), *k* = 10.

## Results

### Sociodemographic characteristics and clinical assessment

Comparisons between group I [*n* = 20; olanzapine (*n* = 7) + clozapine (*n* = 13)] and group II [*n* = 20; amisulpride (*n* = 8) + aripiprazole (*n* = 12)] are presented in Table1. Analyses showed no significant differences between group I and group II in terms of age, gender, premorbid estimated verbal IQ, or education. No significant differences (*χ*^2^ = 3.683 *p* = 0.159) were observed with respect to concomitant treatment with antidepressants (reboxetine and bupropion). As previously reported, groups largely differed in frequency and severity of co-occurring OCS. Whereas only one patient in group II reported at least mild symptom severity (YBOCS ≥ 8) according to interpretation guidelines [52], 14 patients within group I fulfilled this criterion. Of these 14, only 3 reported OCS onset prior to initiation of clozapine or olanzapine medication. Results of the HZI showed that compulsions mainly consisted of checking and counting behavior. Groups did not differ in duration of illness before index treatment, the severity of positive symptoms, depressive symptoms, general psychopathology or the level of psychosocial functioning. Patients in group I tended to show higher overall severity of negative symptoms according to PANSS rating, however, no differences in specific negative symptom domains were found (see SANS in Table 1). No differences in CPZ dosage equivalents were found between the two groups, referring to the following mean (± SD) dosages (clozapine: 348.1 ± 144.5; olanzapine: 16.4 ± 4.8; amisulpride: 425.0 ± 190.9; aripiprazole: 17.5 ± 6.2).

**Table 1:** Between-group differences in sociodemographic and clinical characteristics

	Group I (*n* = 20) Mean ± SD, 95% CI	Group II (*n* = 20) Mean ± SD, 95% CI	Between-group differences	Effect size
Sociodemographics				
Age	40.7 ± 9.8, (36.1, 45.3)	38.9 ± 10.8, (33.8, 44.0)	*T* = 0.551 *p* = 0.585	*d* = 0.17
Male/female ratio	18:2	13:7	*χ*^2^ = 3.584 *p* = 0.058	OR 4.85
Duration of illness before ‘index treatment^a^’ (years)	7.5 ± 8.8, (3.4, 11.6)	5.5 ± 6.6, (2.3, 8.6)	*T* = 0.805 *p* = 0.426	*d* = 0.26
Education (years)	11.5 ± 1.7, (10.7, 12.3)	11.2 ± 1.8, (10.4, 12.0)	*T* = 0.524 *p* = 0.594	*d* = 0.17
Premorbid intelligence	111.5 ± 16.6, (103.7, 119.2)	110.4 ± 12.5, (104.7, 116.9)	*T* = 0.212 *p* = 0.833	*d* = 0.07
Antipsychotic medication				
Duration of index treatment (years)	6.8 ± 4.9, (4.5, 9.1)	1.7 ± 1.9, (0.8, 2.6)	*T* = 4.326 *p* < 0.001	*d* = 1.37
Dosage mg/day (CPZ)	336.7 ± 122.8, (279.3, 394.2)	321.8 ± 124.8, (248.0, 372.0)	*T* = 0.376 *p* = 0.709	*d* = 0.12
Psychopathology				
YBOCS				
Obsessions	6.4 ± 4.7, (4.1, 8.6)	0.3 ± 1.3, (− 0.3, 0.9)	*Z* = − 4.235 *p* < 0.001	*r* = 0.67
Compulsions	6.1 ± 5.4, (3.5, 8.6)	1.4 ± 2.8, (0.0, 2.7)	*Z* = − 2.966 *p* = 0.008	*r* = 0.47
Total	12.4 ± 9.2, (8.1, 16.7)	1.6 ± 3.8, (− 0.2, 3.4)	*Z* = − 3.892 *p* < 0.001	*r* = 0.62
HZI				
Checking	5.1 ± 3.0, (3.7, 6.5)	2.3 ± 2.1, (1.3, 3.3)	*Z* = − 3.040 *p* = 0.002	*r* = 0.48
Washing	1.5 ± 1.7, (0.7, 2.4)	1.6 ± 1.6, (0.8, 2.4)	*Z* = **−** 0.242 *p* = 0.817	*r* = 0.04
Ordering	2.6 ± 1.7, (1.8, 3.5)	2.2 ± 1.5, (1.5, 2.9)	*Z* = **−** 0.688 *p* = 0.506	*r* = 0.11
Counting	2.2 ± 2.4, (1.0, 3.4)	0.5 ± 0.8, (0.5, 0.2)	*Z* = − 2.662 *p* = 0.012	*r* = 0.42
Obsessions	2.3 ± 2.0, (1.3, 3.2)	2.2 ± 1.5, (2.2, 1.4)	*Z* = **−** 0.210 *p* = 0.840	*r* = 0.03
Aggressive obsessions	1.3 ± 1.9, (0.4, 2.2)	0.3 ± 0.7, (− 0.1, 0.6)	*Z* = **−** 1.953 *p* = 0.123	*r* = 0.31
PANSS				
Positive Scale	13.8 ± 3.1, (12.3, 15.2)	13.0 ± 3.0, (11.6, 14.4)	*T* = 0.780 *p* = 0.440	*d* = 0.26
Negative Scale	16.7 ± 4.3, (14.7, 18.7)	13.8 ± 4.4, (11.7, 15.8)	*T* = 2.134 *p* = 0.039	*d* = 0.67
General psychopathology	34.3 ± 4.7, (32.0, 36.5)	32.0 ± 5.4, (29.4, 34.5)	*T* = 1.436 *p* = 0.159	*d* = 0.54
SANS				
Affective flattening	1.6 ± 1.2, (1.0, 2.1)	1.3 ± 1.2, (0.7, 1.8)	*T* = 0.791 *p* = 0.434	*d* = 0.25
Alogia	1.5 ± 1.2, (0.9, 2.1)	1.0 ± 1.1, (0.4, 1.5)	*T* = 1.488 *p* = 0.145	*d* = 0.43
Avolition–apathy	2.0 ± 1.0, (1.6, 2.6)	1.5 ± 1.3, (0.9, 2.1)	*T* = 1.641 *p* = 0.109	*d* = 0.43
Anhedonia	2.0 ± 1.0, (1.5, 2.5)	1.5 ± 1.5, (0.7, 2.2)	*T* = 1.351 *p* = 0.185	*d* = 0.39
Attention	2.0 ± 1.3, (1.3, 2.6)	1.5 ± 1.3, (0.9, 2.1)	*T* = 1.219 *p* = 0.230	*d* = 0.38
CDSS	1.1 ± 1.5, (0.4, 1.8)	1.8 ± 2.8, (0.5, 3.1)	*T* = − 0.993 *p* = 0.327	*d* = 0.31
PSP	67.6 ± 6.5, (64.6, 70.6)	71.1 ± 7.1, (67.7, 70.6)	*T* = − 1.610 *p* = 0.116	*d* = 0.51

*CDSS* Calgary Depression Scale for Schizophrenia, *CI* confidence interval, *CPZ* chlorpromazine equivalents, *HZI* Hamburger Zwangsinventar, *OR* odds ratio, *PANSS* Positive and Negative Syndrome Scale, *PSP* Personal and Social Performance Scale, *SD* standard deviation, *SANS* Scale for the Assessment of Negative Symptoms, *YBOCS* Yale–Brown Obsessive–Compulsive Scale

^a^Treatment with clozapine, olanzapine, aripiprazole or amisulpride

### Functional MRI

#### Behavioral data

The two groups did not significantly differ in their performance in the face-matching task, neither regarding percent of correct answers (*p*s > 0.31) (group I: faces: 95.83 ± 9.70; forms: 97.71 ± 3.70; group II faces: 98.12 ± 2.52; forms: 97.29 ± 3.39) nor regarding reaction times (*p*s > 0.77) (group I: faces: 1619.83 ± 443.30 ms; forms: 1243.08 ± 354.39 ms; group II: faces: 1583.79 ± 322.47 ms; forms: 1230.11 ± 260.61 ms).

#### Brain activation data and connectivity

**Fig. 1:**
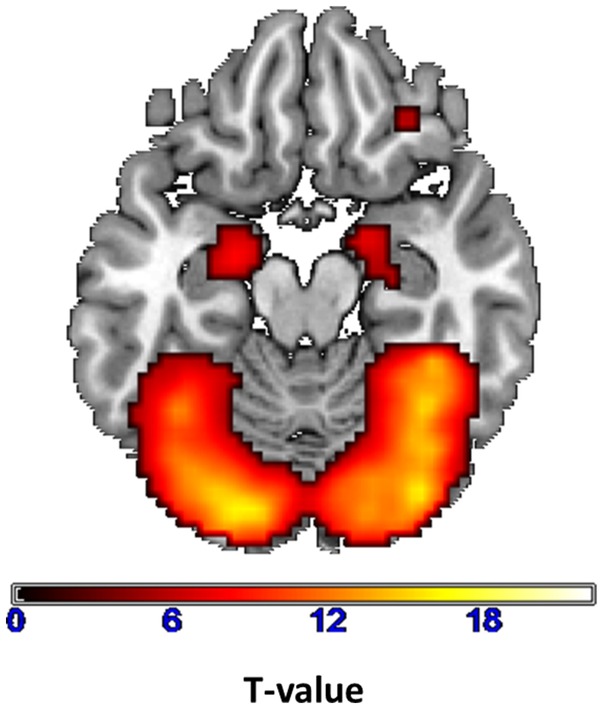
Enhanced activity for faces > geometrical figures (displayed with: *p* < 0.05 FWE corrected, *k* = 10) across all participants

Regarding the main effect of condition in the implicit emotion recognition task, the comparison of faces with geometrical shapes revealed activation in occipital regions, inferior frontal gyrus, thalamus, insula and amygdala (Fig. 1; Table 2). The enhanced amygdala activation was also confirmed by ROI analyses (left coordinates: *x* = − 21, *y* = − 7, *z* = − 18; *T* = 8.13; *p* < 0.001 (svc); *k* = 45; right coordinates: *x* = 21, *y* = − 4, *z* = − 15; *T* = 7.31; *p* < 0.001 (svc); *k* = 43).

**Table 2:** Activity across all participants during face matching (> matching of geometrical shapes; *p* < 0.05 FWE corrected, *k* = 10)

Main effect faces	BA	Cluster	MNI			*T* value
Area			*x*	*y*	*z*	
Middle occipital gyrus	18	5.312	18	− 100	12	20.06
Lingual gyrus	18		6	− 88	− 9	19.68
Middle occipital gyrus	18		36	− 82	− 15	17.28
Thalamus		598	24	− 28	− 3	11.81
Thalamus			− 21	− 31	0	9.92
Amygdala			− 21	− 10	− 15	8.57
Insula	13	415	42	8	27	9.26
Inferior frontal gyrus	46		45	26	18	8.87
Inferior frontal gyrus	45		54	38	9	6.50
Inferior frontal gyrus	47	16	33	32	− 15	6.54
Inferior frontal gyrus	9	20	− 45	14	24	6.13
Inferior frontal gyrus	45	11	− 57	26	21	5.62

**Fig. 2:**
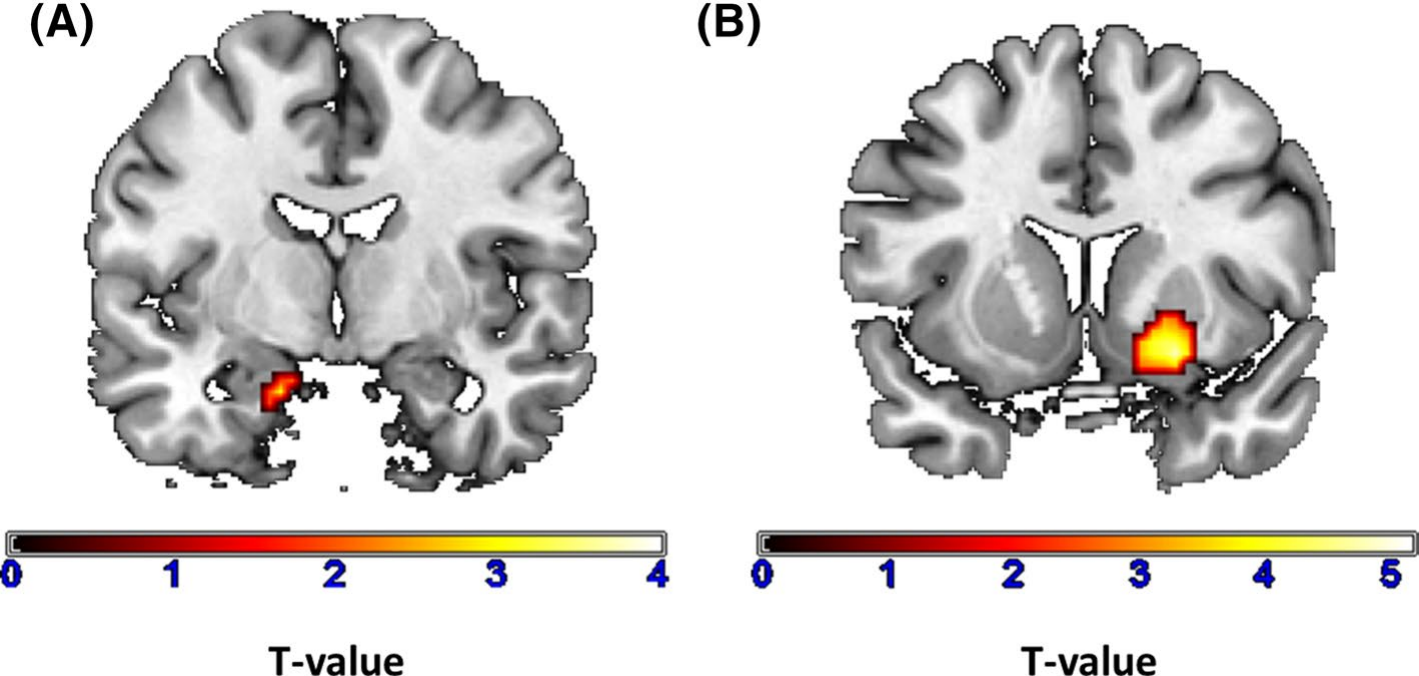
Reduced activity and connectivity of left amygdala in group I (patients treated with clozapine or olanzapine) in comparison to group II (patients treated with aripiprazole or amisulpride). **a** Reduced activity of left amygdala for the contrast faces > geometrical figures. **b** Reduced connectivity of left amygdala to right ventral striatum. Note, activity and connectivity differences between groups are presented unmasked and are displayed with: *p* < 0.005, *k* = 10

Whole brain group comparisons at the given significance threshold (*p* < 0.05 FWE corrected, *k* = 10) revealed no significant group differences. When applying a ROI analysis for the amygdala, we found decreased activity in left amygdala in group I (coordinates: *x* = − 18, *y* = − 4, *z* = − 25; *T* = 2.62; *p* = 0.047 (svc); *k* = 18; Fig. 2a) compared to group II.

Regarding associations between amygdala activation and OCS, regression analyses were conducted. Analyses revealed a significant negative association between obsessive–compulsive severity (YBOCS total score) and bilateral amygdala activation (left coordinates: *x* = − 18, *y* = − 7, *z* = − 17; *T* = 2.98; *p* = 0.023 (svc); *k* = 44; right coordinates: *x* = 18, *y* = − 4, *z* = − 21; *T* = 2.76; *p* = 0.035 (svc); *k* = 42) across groups, as well as within group I (left coordinates: *x* = − 27, *y* = − 7, *z* = − 15; *T* = 3.35; *p* = 0.018 (svc); *k* = 28; right coordinates: *x* = 24, *y* = − 7, *z* = − 15; *T* = 3.34; *p* = 0.020 (svc); *k* = 34).

#### Group comparison connectivity

Whole brain group comparisons at the given significance threshold (*p* < 0.05 FWE corrected, *k* = 10) revealed no significant group differences. When applying ROI analysis, decreased connectivity from left amygdala to right ventral striatum (*x* = 15, *y* = 11, *z* = − 12; *T* = 3.38, *p* = 0.006 (svc); *k* = 25) in group I compared to group II became apparent (Fig. 2b).

## Discussion

### Differential SGA effects on brain activation and connectivity

Analyses of brain activation during the face-matching task revealed attenuated activation in the amygdala in patients treated with clozapine or olanzapine, while no between-group differences were found in task performance on the behavioral level. Furthermore, this group showed decreased connectivity from left amygdala to right ventral striatum. Reduced amygdala activation was associated with higher severity of OCS in the whole sample and within group I.

So far, the number of studies investigating differential effects of antipsychotic substances on brain regions involved in emotional processing is limited. Results from animal studies showed attenuation of amygdala activation under clozapine [54] and differential effects of aripiprazole and olanzapine on 5-HT1A serotonin receptor expression in the limbic system of the rat brain [55]. In humans, both quetiapine and risperidone have been described to increased BOLD-signal in brain regions involved in emotional processing [24]. Our results and these findings support the assumption that the pharmacodynamic fingerprints of SGAs might indeed influence neural activation during emotional processing. However, no previous study accounted for varying receptor profiles of different SGAs.

### Association between amygdala activation and connectivity and OCS

In general, the cortico–limbic interaction plays an important role in theories of emotional processing in OCD [37], in particular the activation and connectivity of the amygdala. Studies have been inconsistent with regard to hyper- vs hypoactivation of the amygdala in primary OCD patients [34]. Increased amygdala activation and task-dependent functional connectivity have been reported during symptom provocation [56, 57], response inhibition [58] and emotional face processing [39]. In contrast, other studies reported less amygdala activation to general emotional stimuli or facial expressions in primary OCD [40, 41].

Beyond important experimental differences and perspectives of comparison, some authors explain the heterogeneity of the results with reciprocal functional relationships between the amygdala and the prefrontal cortex. They hypothesized that the commonly reported hyperactivity of the CSTC found in OCD may dampen amygdala activation to disorder-irrelevant stimuli, such as facial expression [40, 41]. Reduced connectivity between the limbic system and cortico-striatal circuits has been described in antidepressant-free OCD patients at rest [59–61] and during reward tasks [62, 63].

In summary, existing fMRI studies suggest that OCD may be characterized by alterations in amygdala activation and in the interaction between the limbic and cortico-striatal system. Inconsistent results on the direction of these alterations might dependent on the experimental setting and state of the OCD patient. These findings in primary OCD patients together with the negative association between amygdala activation and reported OCS severity in the current sample and specifically within group I suggest that the observed reduced amygdala activation and aberrant connectivity during treatment with mainly antiserotonergic SGAs might represent a neural mechanism involved in the de novo development and maintenance of comorbid OCS in patients with schizophrenia.

### Limitations

This non-interventional study is confined by limitations due to its cross-sectional design and observational nature. This impedes any causal assumptions between the type of antipsychotic medication, aberrations in brain functioning and clinical presentation of OCS. Longitudinal studies with a randomized and blinded design would be necessary to further elucidate proposed causal interrelations. Longitudinal clinical observations of the here described groups showed progressive differences in OCS severity over a 1 year period, supporting the assumption of causal influences [26]. Unfortunately, imaging data were only collected cross-sectionally and sample sizes were relatively small. The stratification of participants into four groups for comparisons of individual substances was therefore not possible due to lack of power. Furthermore, the proposed differential effect of antipsychotic medication is certainly not restricted to alterations within the serotonergic neurotransmission, but reciprocal interactions between serotonergic, dopaminergic and glutamatergic neurotransmission are most likely [64]. Furthermore, groups differed in the duration of treatment. We assume that the longer treatment duration of index medication within group I might represent a component of the proposed causative pharmacodynamics property of SGA treatment. Previous results suggest that especially clozapine aggravates or induces OCS in a dose- and time-dependent manner [25, 65]. In contrast, there is no evidence that duration of other antipsychotic treatment is correlated with severity of OCS in schizophrenia. Other relevant variables such as age, gender, estimated IQ, the severity of psychotic symptoms, general psychopathology and level of psychosocial functioning did not differ between groups. In addition, including a healthy control group for comparison would have strengthened the results. Finally, the updated SPM12 version has several advantages over SPM8 (https://www.fil.ion.ucl.ac.uk/spm/software/spm12/), therefore the use of SPM8 for data analyses is a limitation that should be considered when interpreting the results.

### Conclusion

Aberrant amygdala activation and interaction between the amygdala and other limbic circuits during emotional processing was associated with the type of antipsychotic treatment and severity of co-occurring OCS in patients with schizophrenia. In line with previously reported differences in OFC activation during an inhibitory control task, this finding contributes to a neurobiological theory of SGA-induced or -aggravated OCS. Further studies are needed to investigate the proposed pathogenic pathways and might help to identify risk constellations and early detection of second-onset OCS and hopefully contribute to improved treatment options.
